# A Rare Case of Pyknodysostosis (Toulouse-Lautrec Syndrome): Dental Perspectives on Comprehensive Management

**DOI:** 10.4317/jced.62495

**Published:** 2025-05-01

**Authors:** Rishi Tyagi, Amit Khatri, Deepak Khandelwal, Padma Yangdol, Aman Kumar, Harshita Bisht, Shaikh Misbah, Chithaluru Pranathi, Urvi Bhatia

**Affiliations:** 1 Professor (SAG) & Head, MDS, Department(s) and institution(s): Department of Pediatric and Preventive Dentistry, University College of Medical Sciences, Guru Teg Bahadur Hospital (University of Delhi), Delhi-110095; 2 Professor (SAG), MDS, Department(s) and institution(s): Department of Pediatric and Preventive Dentistry, University College of Medical Sciences, Guru Teg Bahadur Hospital (University of Delhi), Delhi-110095; 3 Assistant Professor, MDS, Department(s) and institution(s): Department of Pediatric and Preventive Dentistry, University College of Medical Sciences, Guru Teg Bahadur Hospital (University of Delhi), Delhi- 110095; 4 Senior Resident, MDS, Department(s) and institution(s): Department of Pediatric and Preventive Dentistry, University College of Medical Sciences, Guru Teg Bahadur Hospital (University of Delhi), Delhi- 110095; 5 Post Graduate Resident MDS, Department(s) and institution(s): Department of Pediatric and Preventive Dentistry, University College of Medical Sciences, Guru Teg Bahadur Hospital (University of Delhi), Delhi- 110095

## Abstract

Pyknodysostosis (PKND), also referred to as Toulouse-Lautrec Syndrome, is a rare autosomal recessive disorder marked by short limbs, short stature, and generalized bone sclerosis. The hallmark signs of this disorder include sclerosis of the terminal phalanges, persistent fontanelles, delayed suture closure, wormian bones, absence of frontal sinuses, obtuse mandibular gonial angle, and relative mandibular prognathism. This case report elucidates a 13-year-old boy presenting with systemic features such as short stature, frontal and parietal bossing, depressed nasal bridge, a beaked nose, hypoplastic midface, wrinkled skin on the fingertips, and nail abnormalities. The oro-dental manifestations include deep palate, prominent palatal rugae, constricted maxillary arch, proclined maxillary anterior teeth and Class III skeletal profile. Radiographic findings showed hypoplastic paranasal sinuses, atrophic mandible, taurodontism, impacted permanent teeth along with several retained deciduous molars. This case highlights the need for vigilance in identifying the dental and systemic signs of PKND, emphasizing the importance of early diagnosis and tailored treatment strategies to improve patient outcomes.

** Key words:**Pyknodysostosis, Toulouse-Lautrec syndrome, Dental management.

## Introduction

Pyknodysostosis is a rare autosomal recessive disorder first described in 1962 by Andren *et al*. (1). The term ‘pyknodysostosis’ originates from Greek literature, where ‘pykno’ means dense, ‘dys’ means defect, and ‘ostosis’ means bone (indicating a dense bone defect) (2). Initially, it was considered an atypical form of cleidocranial dysplasia. Key characteristics of pyknodysostosis include short stature (under 150 cm), widespread diffuse osteosclerosis with a predisposition to fractures from minor trauma, hypoplastic clavicles, and acro-osteolysis accompanied by bone sclerosis (3).

Other noTable features include wrinkled skin, abnormalities of the fingers and nails, kyphosis, scoliosis, a history of recurrent chest infections, and sleep apnea. Patients generally have normal intellectual and sexual development (4).Cranial and maxillofacial characteristics involve frontoparietal bossing, a thickened calvaria, open fontanelles and sutures, hypoplastic paranasal sinuses, Wormian bones in the lambdoidal region, relative proptosis, a beaked nose, a hypoplastic midface, and an obtuse mandibular gonial angle, often accompanied by relative prognathism (5). Bone undergoes constant remodeling process with the help of bone forming cells (osteoblasts) and bone resorbing cells (osteoclasts). Pyknodysostosis is caused by mutation in the Cathepsin K (CTSK) gene. This gene encodes an enzyme called Cathepsin K which is a cysteine type protease that regulates osteoclasts leading to decreased bone resorption (6). This case report details a 13-year-old boy diagnosed clinically with Toulouse-Lautrec syndrome, highlighting his dental manifestations.

## Case Report

**Figure 1:**
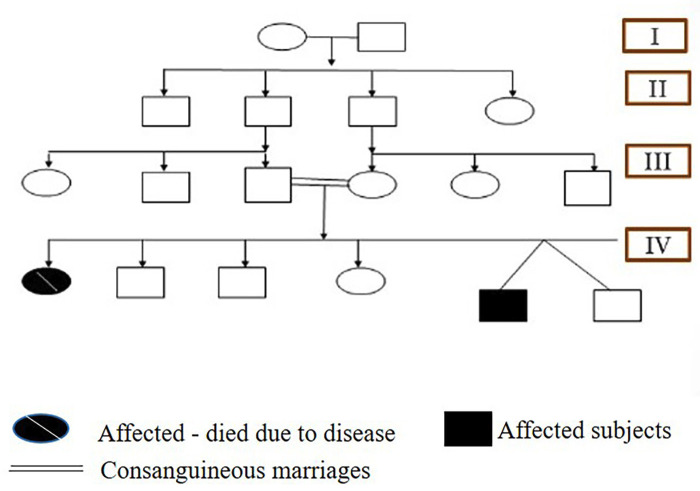
Pedigree chart representing four generations of the individuals affected with pyknodysostosis. Filled symbols identify affected subjects, filled symbols with slash depicts deceased affected individual. Consanguineous marriages are represented with double lines.

**Figure 2:**
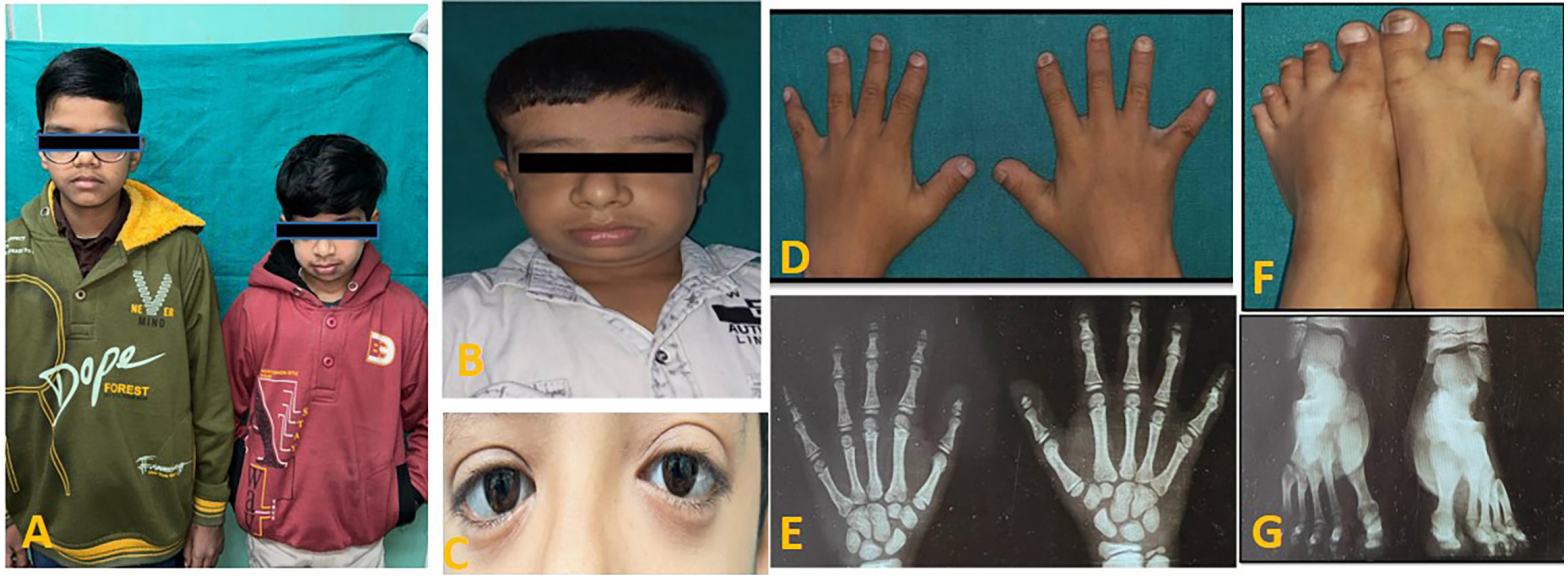
A to G: (A) Non-identical twins in which one is affected and the other is normal (B) Extraoral view. (C) Bluish sclera and hypertelorism (D) Short, stubby fingers-Brachydactyly (E)Antero-posterior radiograph of the hands showing acro osteolysis (F)Short feet with distal phalanges short (G)Antero-posterior radiograph of the feet showing sandal gap deformity and acro osteolysis.

**Figure 3:**
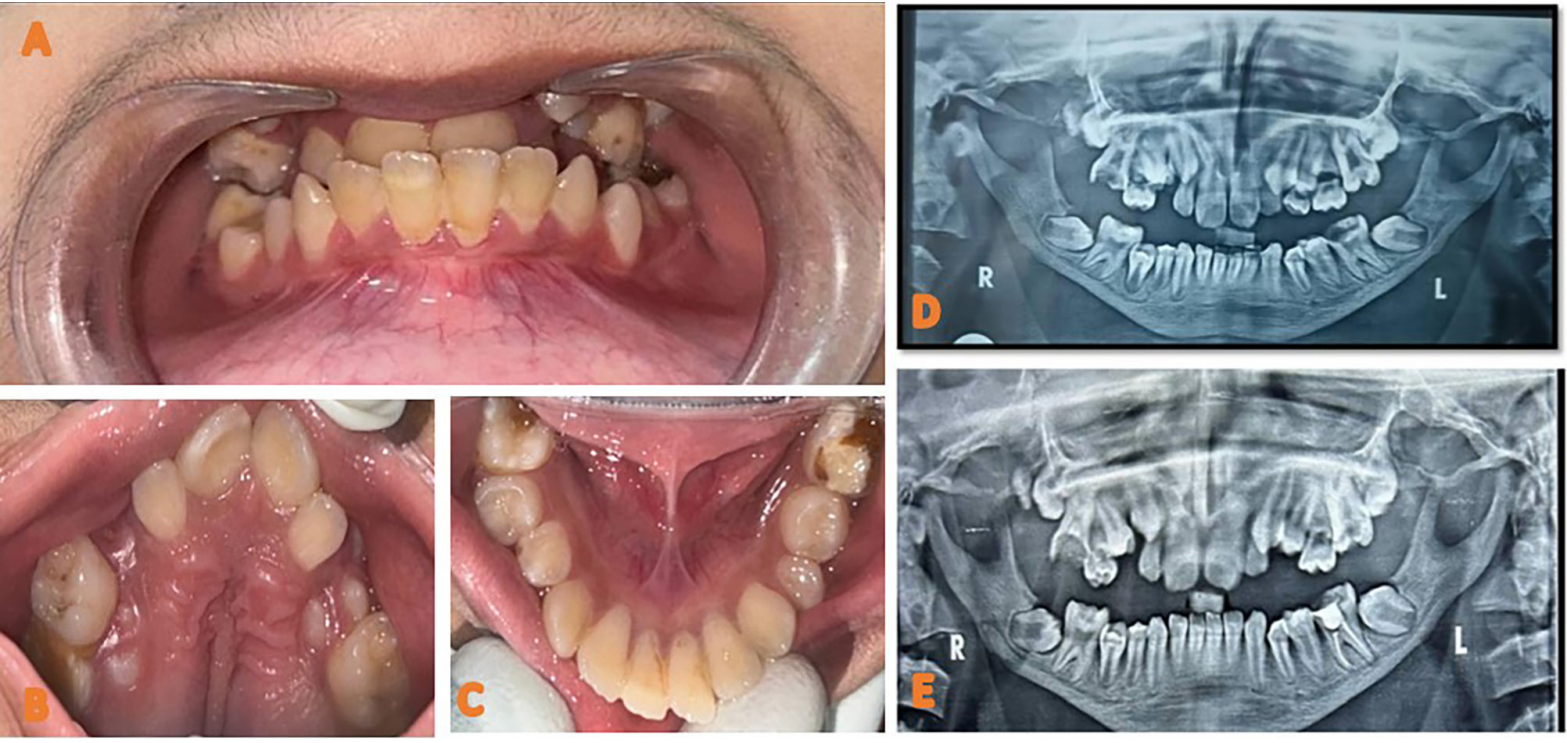
A and E: (A) Intraoral picture depicting anterior and posterior crossbite. (B) Intraoral view of maxilla showing prominent palatal rugae, deep palate, crowded upper anteriors and constricted maxillary arch (C)Intraoral view of mandible showing proclined mandibular incisors, dental caries in relation to left and right lower first permanent molar. (D) Preoperative orthopantomogram showing dental caries in relation to 16 and 36 , hypoplastic jaw, taurodontism impacted permanent teeth and retained deciduous teeth (E) Postoperative orthopantomogram showing Root canal therapy done with respect to 36 and MTA pulpotomy done with respect to 16 followed by restoration with Glass Ionomer Cement.

A 13-year-old male patient reported to the department of Pediatric and preventive dentistry University of Medical Sciences, Guru Tegh Bahadur (GTB) hospital with a chief complaint of pain in his left lower back tooth region since 20 days. The patient’s past history was noTable for disappropriate short stature and left sided focal seizures. Additionally, the patient gives history of preterm vaginal birth. The patient also gives history of fall leading to multiple skull bone fracture and small haemorrhagic contusion in left temporal brain parenchyma. Family history was significant for consanguineous parents. Parents gave birth to non-identical twins with one child affected by the disease and the other child normal summarized in pedigree chart (Fig. 1). On physical examination, the patient had disappropriate short stature with a height of 116 cm (<3rd percentile), weight of 18.2 kgs (<3rd percentile), body mass index of 13.52 (<5th percentile), and head circumference of 46 cm. Further general examination showed fronto -parietal bossing, disappropriation of size of his head with face which was small, prominent nose with convex nasal bridge, absence of prominence of zygomatic bone, bluish sclera, hypoplastic midface, anterolateral bowing of legs, short, stubby fingers (brachydactyly), thick lower lips and obtuse mandibular angle with mandibular prognathism. Hand wrist radiographs showed mid phalangeal acrolysis on the distal phalanges (Fig. 2). Intraoral clinical examination revealed anterior and posterior crossbite, buccally placed left maxillary molar, deep palate, crowded upper anteriors , constricted maxillary arch and proclined mandibular incisors, dental caries in relation to left lower first permanent molar. The patient had skeletal and dental class III profile (Fig. 3). Lateral teleradiography showed thickening of the cranial base, hypoplastic mandible with obtuse mandibular angle, cortical thickening and elongation of coronoid apophyses and mandibular condyles. Three-dimensional CT image revealed open fontanelles and sutures, wormian bones in the lambdoid region. Panoramic radiography showed thin mandible with crowding of maxillary teeth and delayed eruption of permanent teeth was noted ( Table 1).

The patient exhibited no signs of mental deficiency and was with a normal Intelligence Quotient as compared to the children of his age group. The laboratory findings revealed decreased creatinine (0.3mg/dl), low calcium (9.9mg/dl) high phosphorus (5.5mg/dl), increased AST (49 U/L), increased ALP (460U/L) and decreased globulin (2.6 g/dl) ( Table 1). A request was made for genetic testing for cathepsin K gene, which revealed a genetic variant on chromosome 1.150771644C>G. and a specific variant in the CTSK (NM000396.4)C.890G>C. According to American College of Medical Genetics and Genomics(ACMG), the variant was found to be pathogenic. An association of clinical, laboratory, genetic and radiographic data suggested pyknodysostosis as the most likely diagnosis ( Table 1). The dental management was done according to the symptoms presented at the time of examination. Patient presented with a non-vital tooth with respect to 36, sharp pain with respect to 16. Root canal therapy was done with respect to 36 and, MTA pulpotomy was done with respect to 16 followed by restoration with Glass Ionomer Cement.

## Discussion

Pyknodysostosis was first identified in 1962 and is a rare autosomal recessive skeletal disorder with full penetrance. Fewer than 200 cases have been reported in the literature, and its estimated prevalence ranges from 1 to 1.7 per million. The disorder affects both males and females equally, and consanguinity is noted in about 30% of cases (7). The clinical features were initially described in 1962 by Maroteaux and Lamy, as well as Andren *et al*. (8). The French artist Henri de Toulouse-Lautrec was posthumously thought to have had this condition, although this diagnosis has recently been questioned (8). The three key characteristics of Pyknodysostosis are disproportionate short stature, increased bone density (osteosclerosis), and acro-osteolysis (bone resorption at the fingertips). Disproportionate short stature often serves as the first sign of the disorder, prompting further investigation. Osteosclerosis is present in all affected individuals, and while it is visible on radiographs at the time of diagnosis, its clinical impact can vary. The average fracture rate is moderate, with one study reporting 0.2 fractures per year. However, many patients report no fractures, even into adulthood. The diagnosis of Pyknodysostosis (PKND) is often confirmed through the presence of diffuse sclerosis, cortical thickening, and acro-osteolysis on plain radiographs, alongside other common clinical features. Acro-osteolysis is the second most frequent skeletal abnormality identified at diagnosis in most cases. This distinctive radiographic sign is highly prevalent in PKND patients, underscoring the importance of thorough radiographic evaluation in individuals showing signs of sclerosing bone disorders. In the case of our patient, initial radiographic analysis of the skull, hands, and feet by a radiologist provided sufficient evidence for diagnosing PKND. Other notable features observed included open fontanelles, delayed fusion of skull sutures, and hypoplastic maxillary and paranasal sinuses.

In addition to disproportionate short stature, PKND patients commonly exhibit distinct facial characteristics and dental abnormalities. While not every feature is seen in every patient, about half of individuals with PKND present with these traits. The most frequent facial anomaly is micrognathia, followed by frontal bossing, mandibular hypoplasia, aquiline nose, proptosis, maxillary hypoplasia, and blue sclera (9).

Individuals with pyknodysostosis often present with several dental challenges, including:

1. Delayed Tooth Eruption: Primary and permanent teeth may erupt later than normal.

Tooth eruption can be disordered, with some teeth appearing in an unusual order or position.

2. Malocclusion: Due to skeletal abnormalities, people with pyknodysostosis often have malocclusion (misalignment of the teeth and bite), which can complicate dental care and oral hygiene (10).

3. Increased Risk of Tooth Fractures: Fragile bones can lead to a higher risk of dental fractures and trauma to the teeth. This includes both primary (baby) and permanent teeth (11).

4. Decreased Bone Density in the Jaw: Pyknodysostosis is associated with low bone density, which can affect the jawbones and complicate procedures like extractions or implants. Periodontal disease can be more severe, and bone resorption may happen faster (12).

5. Crowded Teeth: The size and shape of the jaw may contribute to crowding and improper alignment of the teeth, which could necessitate orthodontic treatment.

6. Enamel Defects: There might be abnormalities in tooth enamel, making the teeth more susceptible to decay or damage (13).

As of now, no specific guidelines have been documented in the literature; however, the general precautions to follow when handling a case of pyknodysostosis are:

1. Early Intervention: Regular dental check-ups should begin early in life, even before the age of 1, to monitor the progression of tooth eruption, jaw development, and potential dental issues. Early orthodontic evaluation may be needed to address issues with malocclusion or crowding, but any intervention should be planned carefully due to fragile bones.

2. Gentle Handling During Dental Procedures: Due to brittle bones, gentle handling during dental visits is important to prevent fractures or injury, especially when using forceps or during tooth extractions.

3. Preventive Care: Regular fluoride treatments can help protect teeth from decay, especially if enamel defects are present. Sealants may be applied to protect vulnerable surfaces of the teeth from cavities. Emphasis on oral hygiene practices (e.g., brushing, flossing) to prevent plaque buildup and gum disease, which can be more pronounced in people with pyknodysostosis due to weakened bone structure (14).

4. Management of Tooth Fractures: For individuals prone to tooth fractures, protective mouthguards should be considered, especially for children involved in physical activities. Restorative dental care (e.g., crowns, fillings) might be necessary for fractured teeth or to protect teeth from further damage (10).

5. Considerations for Tooth Extractions: If extractions are needed, care should be taken to ensure that the bone density and fragile nature of the jaw are taken into account. Post-extraction care should focus on wound healing and preventing infections. Bone grafting might be necessary for severe bone resorption in the jaw (15).

6. Potential for Orthodontic Treatment: Orthodontic interventions should be planned with extreme caution, considering the risk of fractures. Techniques like expanders or braces may be used but should be approached carefully to avoid trauma to the jaw (10).

7. Interdisciplinary Care: Collaboration with geneticists, orthodontists, and oral surgeons is essential for comprehensive care. They can help monitor the development of the condition and ensure that treatments are appropriate for the patient’s bone health (16).

A key aspect of our patient’s case was the positive family history, as consanguinity is documented in many PKND cases, particularly in regions where such relationships are more common (17). There is currently no specific treatment for PKND, and management remains primarily supportive (18). Due to the risk of fractures, efforts should focus on minimizing fracture risks (19). Complications such as mandibular fractures and osteomyelitis should be considered during minor surgeries like tooth extraction (4). Children with PKND should receive specialized dental care, including preventive treatments and ongoing monitoring of growth and craniofacial development. Overall, the prognosis for individuals with pyknodysostosis is generally favorable, with no significant systemic complications, and life expectancy is typically normal.

## Conclusions

The diagnosis of Pyknodysostosis (PKND) is primarily based on clinical and radiographic findings, though genetic testing is essential for confirming the diagnosis (20). Management should involve a multidisciplinary approach to address the symptoms and enhance the patient’s quality of life. It is important to consider potential complications, such as mandibular fractures and osteomyelitis, particularly during minor procedures like tooth extractions or dental implants, as osteomyelitis can be difficult to treat due to the presence of osteosclerosis.

## Figures and Tables

**Table 1:** Key characteristics depicting the systemic and dental manifestations of Pyknodysostosis presented in this case report.

SYSTEMIC MANIFESTATIONS	
Clinical Findings:	Radiographic Findings
Short stature	Generalised progressive osteosclerosis
Fronto parietal bossing	Acro osteolysis of the terminal phalanges
Prominent nose with convex nasal bridge, Absence of prominence of zygomatic bone	Delayed fusion of cranial sutures
	Obtuse mandibular angle due to loss of normal mandibular (gonial angle)
Bluish sclera	
Hypoplastic midface	
Hypertelorism	
Anterolateral bowing of legs	
Short, stubby fingers(brachydactyly)	
Thick lower lips	
Obtuse mandibular angle with mandibular prognathism	
DENTAL MANIFESTATIONS	
Clinical Findings	Radiographic Findings
Anterior and posterior crossbite	Thin mandible
Buccally placed left maxillary molar	Taurodontism
Prominent palatal rugae	Impacted permanent teeth
Deep palate	Retained deciduous teeth
Crowded upper anteriors	
Constricted maxillary arch	
Proclined mandibular incisors	
Dental caries in relation to left lower first permanent molar.	
Skeletal and dental class III profile	
LABORATORY FINDINGS	
Decreased creatinine (0.3mg/dl)	
Serum Calcium (9.9mg/dl)	
High phosphorus (5.5mg/dl)	
Increased AST (49 U/L)	
Increased ALP (460U/L)	
Decreased globulin (2.6 g/dl)	

## Data Availability

The datasets used and/or analyzed during the current study are available from the corresponding author.

## References

[1] Valdes-Flores M, Hidalgo-Bravo A, Casas-Avila L, Chima-Galan C, Hazan-Lasri EJ, Pineda-Gomez E (2014). Molecular and clinical analysis in a series of patients with pyknodysostosis reveals some uncommon phenotypic findings. Int J Clin Exp Med.

[2] Maroteaux P, Lamy M (1962). Pyknodysostosis. Presse Med.

[3] Ramaiah KK, George GB, Padiyath S, Sethuraman R, Cherian B (2011). Pyknodysostosis: report of a rare case with review of literature. Imaging Sci Dent.

[4] Schmitz JP, Gassmann CJ, Bauer AM, Smith BR (1996). Mandibular reconstruction in a patient with pyknodysostosis. J Oral Maxillofac Surg.

[5] Hunt NP, Cunningham SJ, Adnan N, Harris M (1998). The dental, craniofacial, and biochemical features of pyknodysostosis: a report of three new cases. J Oral Maxillofac Surg.

[6] Singh AR, Kaur A, Anand NK, Singh JR (2004). Pyknodysostosis: visceral manifestations and simian crease. Indian J Pediatr.

[7] Andren L, Dymling JF, Hogeman KE, Wendeberg B (1962). Osteopetrosis acro-osteolítica: A syndrome of osteopetrosis, acro-osteolysis and open sutures of the skull. Acta Chir Scand.

[8] Inaoka T, Bilbe G, Ishibashi O, Tezuka K, Kumegawa M, Kokubo T (1995). Molecular cloning of human cDNA for cathepsin K: novel cysteine proteinase predominantly expressed in bone. Biochem Biophys Res Commun.

[9] Bizaoui V, Michot C, Baujat G, Amouroux C, Baron S, Capri Y (2019). Pyknodysostosis: Natural history and management guidelines from 27 French cases and a literature review. Clin Genet.

[10] Khoja A, Fida M, Shaikh A (2015). Pycnodysostosis with Special Emphasis on Dentofacial Characteristics. Case Rep Dent.

[11] Ortegosa MV, Bertola DR, Aguena M, Passos-Bueno MR, Kim CA, de Faria ME (2014). Challenges in the orthodontic treatment of a patient with pycnodysostosis. Cleft Palate Craniofac J.

[12] Fleming KW, Barest G, Sakai O (2007). Dental and facial bone abnormalities in pyknodysostosis: CT findings. Am J Neuroradiol.

[13] O'Connell AC, Brennan MT, Francomano CA (1998). Pycnodysostosis: orofacial manifestations in two pediatric patients. Pediatr Dent.

[14] Jawa A, Setty JV, Vijayshankar LV, Srinivasan I (2020). Pyknodysostosis: Report of a Rare Case and its Dental Management. Int J Clin Pediatr Dent.

[15] El-Mahallawy Y, Sweedan AO, Al-Mahalawy H (2021). Pycnodysostosis: a case report and literature review concerning oral and maxillofacial complications and their management. Oral Surg Oral Med Oral Pathol Oral Radiol.

[16] LeBlanc S, Savarirayan R (2020). Pycnodysostosis. 2020 Nov 5 [updated 2023 Apr 6]. In: Adam MP, Feldman J, Mirzaa GM, Pagon RA, Wallace SE, Amemiya A, editors. GeneReviews® [Internet].

[17] Arman A, Bereket A, Coker A, Kiper PÖ, Güran T, Özkan B (2014). Cathepsin K analysis in a pyknodysostosis cohort: demographic, genotypic and phenotypic features. Orphanet J Rare Dis.

[18] Tolar J, Teitelbaum SL, Orchard PJ (2004). Osteopetrosis. N Engl J Med.

[19] Sedano HD, Gorlin RJ, Anderson VE (1968). Pyknodysostosis-clinical and general considerations. Am J Dis Child.

[20] Schmidt GS, Schacht JP, Knee TS, Shakir MKM, Hoang TD (2020). Pyknodysostosis (Osteopetrosis Acro-Osteolytica). AACE Clin Case Rep.

